# Estimation of Genetic Parameters of Body Weight and Body Size in Different Stages of Pishan Red Sheep

**DOI:** 10.3390/ani16101569

**Published:** 2026-05-21

**Authors:** Nuerabudula Wujiwaili, Younusi Huojiaabudula, Bijiguli Sataer, Ayiguzili Rouzimaimaiti, Gao Gong, Aladaer Qi, Yiming Sulaiman

**Affiliations:** College of Animal Science, Xinjiang Agricultural University, Urumqi 830052, China; norabla2017@outlook.com (N.W.); 18154997428@163.com (Y.H.); bijidigul@outlook.com (B.S.);

**Keywords:** Pishan Red sheep, body weight and body size, genetic parameters, growth traits, 0~12 months of age

## Abstract

As a dual-purpose (meat and fat) coarse-wool sheep breed in Xinjiang, the Pishan Red sheep exhibits year-round estrus and excellent reproductive performance. The aim of this study was to systematically estimate the genetic parameters of growth traits in Pishan Red sheep at each stage from 0 to 12 months of age, and to provide a scientific basis for optimizing early selection indices for this breed. Year of birth, type of birth, and sex were included as fixed effects, while maternal genetic effects and individual additive genetic effects were treated as random effects. A multi-trait animal model was constructed using DMU software to analyze the genetic parameters. The results showed that for growth traits at 0 months of age, heritability ranged from 0.09 to 0.64, genetic correlations from –0.03 to 0.84, and phenotypic correlations from 8.2 × 10^−3^ to 0.32. Among these, the direct heritability of body weight was 0.18, and the maternal heritability was 0.21, while cannon circumference was identified as a high-heritability trait. At 2 months of age, heritability ranged from 0.04 to 0.69, genetic correlations from −0.78 to 0.96, and phenotypic correlations from 0.036 to 0.44. Body weight, body length, and cannon circumference were high-heritability traits. At 6 months of age, heritability ranged from 0.04 to 0.71, genetic correlations from −0.32 to 0.70, and phenotypic correlations from −0.13 to 0.54. Body weight was a medium-heritability trait, while chest width and cannon circumference were high-heritability traits. At 12 months of age, heritability ranged from 0.07 to 0.48, genetic correlations from −0.04 to 0.87, and phenotypic correlations from 0.28 to 0.72. Body weight was a medium-heritability trait, whereas body height, body length, cannon circumference, and heart girth were high-heritability traits. Both body weight and cannon circumference exhibited high heritability throughout the 0–12 month period and can therefore serve as key selection indices for early breeding in this breed. The findings of this study provide essential local baseline data for the genetic improvement, breeding program design, and genetic evaluation of Pishan Red sheep.

## Simple Summary

The Pishan Red sheep is an indigenous meat-and-fat coarse-wool sheep breed from Hotan, Xinjiang, and represents a vital local genetic resource supporting the mutton industry in southern Xinjiang. However, systematic research on its growth traits from 0 to 12 months of age remains insufficient, and reliable early selection indices have not yet been established. In this study, genetic parameters of body weight and body size at multiple ages were estimated using a multi-trait animal model. The results revealed that body weight and heart girth exhibited high heritability throughout the 0–12 month period, with moderate-to-strong genetic and phenotypic correlations among growth traits at different stages. These findings identify body weight and heart girth as key indicators for early selective breeding. The estimated genetic parameters provide essential data for genetic improvement and systematic breeding programs, supporting the conservation and sustainable utilization of local sheep germplasm, and enhancing productivity and economic efficiency in the meat sheep sector of southern Xinjiang.

## 1. Introduction

The Pishan Red sheep is a unique dual-purpose (meat and fat) coarse-wool breed native to the Hotan region of Xinjiang. It exhibits year-round breeding, stable genetic performance, strong resistance, high meat yield, and excellent meat quality, making it a core local germplasm resource for the meat sheep industry in southern Xinjiang. Body size and weight indicators directly reflect body structure, body size, growth, and development, and are closely related to physiological functions, production performance, and adaptive capacity. Therefore, they serve as important phenotypic selection indices in meat sheep breeding [1]. In sheep breeding and production, growth traits such as body weight and body size are influenced by multiple factors, including breed, year of birth, type of birth, sex, and feeding management. Among these, non-genetic factors such as birth month and nutrition can be artificially regulated to improve production performance. Accurate estimation of genetic parameters is the core foundation for scientific breeding [2,3,4].

Currently, systematic genetic parameter studies on growth traits have been conducted in China for breeds such as the Luzhong meat sheep, Alpine Merino, and Hu sheep. However, no systematic genetic parameter estimation of body weight and body size from 0 to 12 months of age has yet been reported for the native Pishan Red sheep in Xinjiang, and the core indices for its early selection and breeding remain unclear. A scientific genetic improvement program can effectively reduce breeding costs and accelerate genetic progress. Accurate estimation of genetic parameters for growth traits—key economic traits in meat sheep breeding—can significantly improve selection accuracy and enhance the overall efficiency and profitability of sheep production systems [5,6]. This study is the first to systematically investigate genetic parameters of body weight and body measurements in Pishan Red sheep from 0 to 12 months of age, estimating heritability, genetic correlations, and phenotypic correlations, and screening core indices for early selection. The aim is to provide data support for the development of scientific breeding management programs and genetic improvement strategies for Pishan Red sheep, to help improve the quality of the meat sheep industry in southern Xinjiang, and to fill the data gaps for local breeding of this breed.

## 2. Materials and Methods

### 2.1. Data Collection and Organization

The experimental data were obtained from the genealogy and production performance measurement data of Pishan Red Sheep from 2021 to 2024 from Xinjiang Hotan Area West Region Muyangren Agricultural and Animal Husbandry Science and Technology Co. The raw data were sorted out using Excel 2019, and after eliminating the data with missing genealogy, abnormal phenotypes, and incomplete measurement records. A total of 16,808 records of body weight and body size data were collected from 5836 Pishan Red Sheep (among them, 5836 records at 0 months of age, 4785 records at 2 months of age, 3691 records at 6 months of age, and 2496 records at 12 months of age), with a genealogy completeness rate of 92.3%, and the genealogy files of 0-, 2-, 6-, and 12-month-old body weight and size were integrated for the subsequent analysis of the genetic parameters.

### 2.2. Measurement Indexes and Methods

Referring to the principle of determination of Livestock Breeding, 8 indices of body weight, body height, body length, chest circumference, chest width, chest depth, tube circumference, and waist angle width of Pishan Red sheep at 0, 2, 6, and 12 months of age were determined. Body weight was measured by electronic scales of 30 kg (0-month-old) and 300 kg (2-, 6-, and 12-month-old) with an accuracy of 0.05 kg, and body size was measured by a soft ruler with an accuracy of 0.1 cm. The GLM module of SAS 9.2 software was used to analyze the ANOVA of body weight and 7 body size indexes, and Duncan’s new complex polarity method was used for multiple comparisons between different levels of each factor. The model of multiple comparisons is shown below:

*Y_jkli_* = *μ* + *m_j_* + *s_k_* + *f_l_* + *e_jkli_*

where *Y_jkli_* is the phenotypic value of the trait; *μ* is the mean value of the trait; *m_j_* is the effect at the *j*-th level of year of birth; *s_k_* is the effect at the *k*-th level of birth type; *f_l_* is the effect at the *l*-th level of sex; and *e_jkli_* is the random error.

### 2.3. Data Organization and Analysis Methods

Descriptive statistics of the complete experimental data were performed using SPSS Statistics version 27.0 (IBM Corp., Armonk, NY, USA). In order to better characterize the effects of the three non-genetic factors selected for this experiment on the body weight and body size indexes of Pishan Red sheep at different stages, the influencing factors of the target traits and the multilevel division of each factor are shown in Table 1 below:

### 2.4. Establishment of Run Files

DMUv6-R5-2-EM software (Aarhus University, Aarhus, Denmark) was used to estimate genetic parameters of growth traits in Pishan Red sheep, and establish three types of core operation files: ① pedigree file (pedigree), containing individual, sire, dam ear number and individual birth date; ② phenotype data file (data record), containing individual number, sire number, dam number, fixed effects and phenotype values of each trait, with missing values of traits indicated by “−99”; and ③ run file (DIR), integrating basic information of individuals and all fixed effects information. The test sheep were numbered uniformly, and the missing parent number was indicated by 0.

### 2.5. Genetic Parameter Estimation Model

According to the multi-trait animal model, the DMUAI module in DMUv6-R5-2-EM software was used to estimate the variance components, and combined with the characteristics of the significant maternal effect during the lactation period of lambs, the maternal genetic effect was set for 0 and 2 months of age, and only the individual additive genetic effect was taken into account for 6 and 12 months of age to construct the 2 types of animal models. The model details are shown in Table 2.

## 3. Results

### 3.1. Analysis of Variance of Body Weight and Body Size Indexes in Different Stages of Pishan Red Sheep

The descriptive statistical results for body weight and body size traits at different months of age are presented in Supplementary Tables S1–S4. In general, the coefficients of variation at 0 and 2 months of age were higher than those at subsequent stages, indicating greater phenotypic variation during the early growth phase.

As shown in Table 3, sex had a significant effect on the growth traits of Pishan Red sheep at 0, 2, 6, and 12 months of age (*p* < 0.05). Birth type had significant effects on body weight, body height, body length, heart girth, chest width, and chest depth at 0, 2, 6, and 12 months of age (*p* < 0.01). Year of birth had significant effects on body weight, body height, body length, heart girth, chest depth, and waist angle width at 0, 2, 6, and 12 months of age (*p* < 0.01).

### 3.2. Estimation of Heritability of Growth Traits in Pishan Red Sheep

Because lambs are greatly influenced by maternal effects during the lactation stage, this study calculated only the maternal heritability of growth traits at 0 and 2 months of age. As shown in Table 4, the maternal heritability ranges for body weight and body size traits of Pishan Red sheep at 0 and 2 months of age were 0.05–0.60 and 0.04–0.69, respectively, with cannon circumference showing the highest maternal heritability (0.64). The traits with the highest heritability at 0, 2, 6, and 12 months of age in Pishan Red sheep were cannon circumference (0.60), body weight (0.69), cannon circumference (0.71), and body height (0.48), respectively. The heritability of body weight first increased and then decreased (ranging from 0.18 to 0.69), remaining within the medium to high heritability range (h^2^ ≥ 0.1).

### 3.3. Genetic Correlation Analysis of Growth Traits in Pishan Red Sheep

As shown in Table 5, the genetic correlations between body size traits and body weight in Pishan Red sheep at 0 months of age ranged from −0.15 to 0.84. Except for the negative genetic correlations between cannon circumference and chest depth, and between waist angle width and body weight, all other pairwise trait combinations—including body height, body length, chest width, chest depth, and cannon circumference—exhibited positive genetic correlations. At 2 months of age, the genetic correlation range was −0.78 to 0.96. With the exception of the following pairs—body length with body weight, heart girth with body height, chest depth with chest width, cannon circumference with chest depth, waist angle width with chest width, and chest depth with cannon circumference—all other trait combinations showed positive genetic correlations. At 6 months of age, the genetic correlation range was −0.32 to 0.70. Except for the pairs of cannon circumference with chest depth and waist angle width with cannon circumference, all other trait combinations showed positive genetic correlations. At 12 months of age, the genetic correlation range was −0.04 to 0.87. Except for the pair of waist angle width with chest width, all other trait combinations exhibited positive genetic correlations.

### 3.4. Phenotypic Correlation Analysis of Growth Traits in Pishan Red Sheep

**Figure 1:**
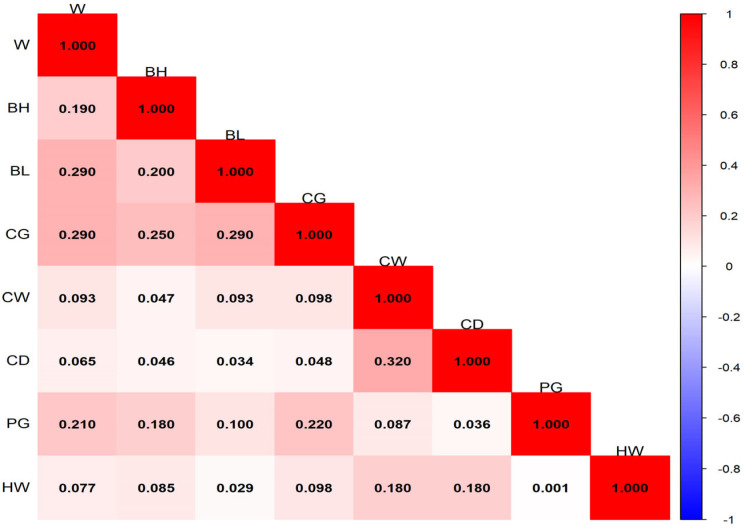
Phenotypic correlation of 0-month-old Pishan Red sheep.

**Figure 2:**
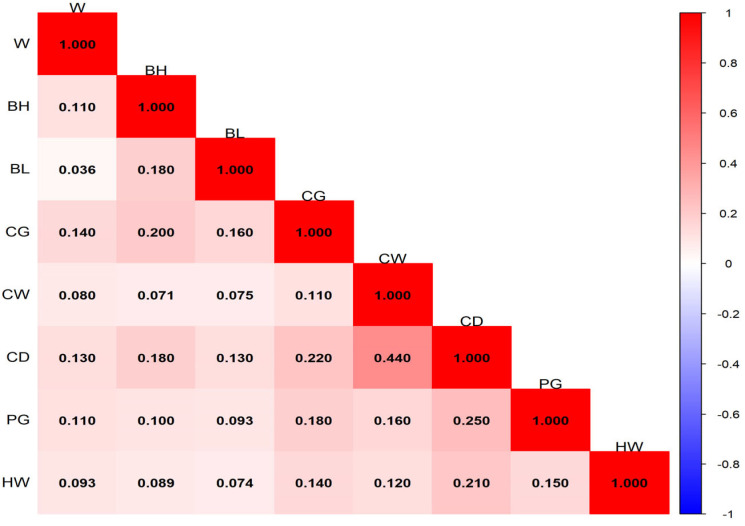
Phenotypic correlation of 2-month-old Pishan Red sheep.

**Figure 3:**
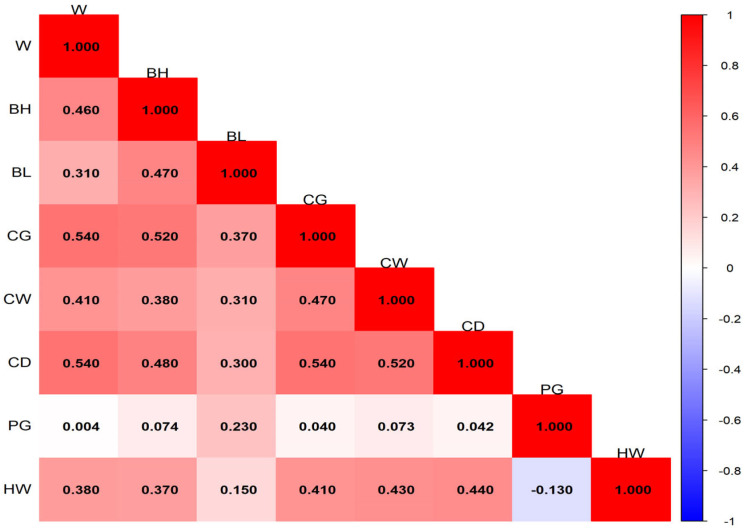
Phenotypic correlation of 6-month-old Pishan Red sheep.

**Figure 4:**
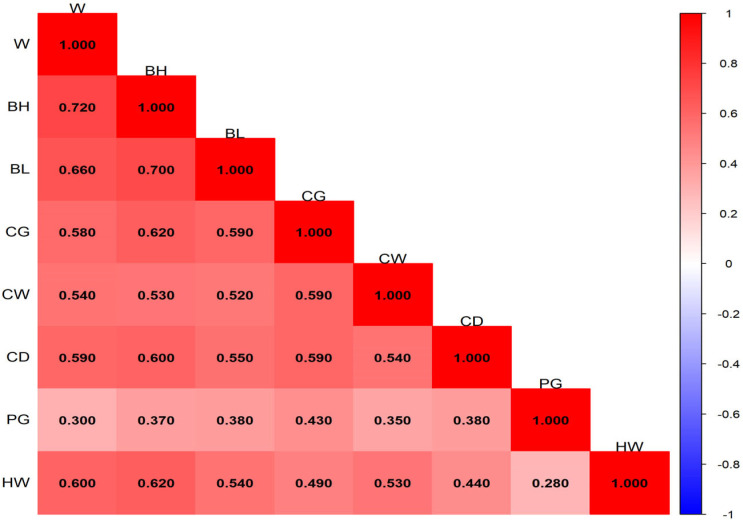
Phenotypic correlation of 12-month-old Pishan Red sheep.

The ranges and distributions of phenotypic correlation coefficients among traits at different months of age are shown in Figure 1, Figure 2, Figure 3 and Figure 4. In general, phenotypic correlations exhibited trends similar to those of genetic correlations, though the values were generally lower. At 12 months of age, the phenotypic correlations between traits were markedly stronger. For example, the correlation coefficient between body height and body length reached 0.72.

## 4. Discussion

### 4.1. Analysis of Heritability of Growth Traits in Pishan Red Sheep

#### 4.1.1. Analysis of Body Weight and Maternal Heritability in Pishan Red Sheep

Heritability is a core indicator of the ability to transmit traits genetically and is also an important basis for determining breeding methods and selection intensity. It is generally considered that heritability <0.2 is low heritability, 0.2~0.4 is medium heritability, and >0.4 is high heritability. In the present study, it was concluded that the heritability of 0-month-old body weight and maternal heritability of Pishan Red sheep were 0.18 and 0.21, respectively, which belonged to medium heritability, and this result was in line with the results of Ren Y et al. [7] for Luzhong meat sheep (0.18 at first birth, 0.20 on the mother), El Fadili M et al. [8] for Timahdit sheep (0.18 at first birth), Mandal A et al. [9] for Muzaffarnagari sheep (neonate 0.21, dam 0.18), but lower than Habtegiorgis K et al. [10] Doyogena et al. sheep (neonate 0.33), Li WH et al. [11] on Alpine Merino sheep (neonate 0.30), and this difference may be related to the genetic background of the breed, rearing and sample size. Related.

The heritability for body weight at 2 months of age and maternal heritability of Pisan Red sheep amounted to 0.69 and 0.19, respectively, with direct heritability being a highly heritable trait and maternal heritability being a moderately low heritability. This is significantly higher than the results of Haile et al. [12] for Sabi sheep (weaning weight 0.13), Rashidi et al. [13] et al. for Kermani sheep (weaning weight 0.27), and Hanford K. J. et al. [14] for Australian Merino sheep (weaning weight 0.10). The significant difference between this result and the heritability of weaning weight of most sheep breeds (usually 0.13 to 0.27) mainly stems from the breed characteristic of excellent year-round and ewe performance of Pishan Red sheep, which makes the growth of lambs during lactation genetically dominated and maternal interference diminished, and which reflects the breed characteristic of fast early growth in this breed.

The heritability of the 6-month-old body weight of Pishan Red sheep was 0.26, which is medium heritability, and usually h^2^ = 0.2~0.4 is the range of medium heritability. Maternal heritability was not calculated because lambs are significantly affected by maternal effects such as performance and lactation behavior during lactation period (0–2 months of age), but at 6 months of age lambs are completely weaned and have independent nutritional sources, so the influence of maternal effects on growth traits has been substantially reduced; therefore, the present study did not set up a model of maternal heritability effects for the 6-month-old stage, and did not estimate the maternal heritability. Ila S. K. et al. [15]. Conducted genetic estimation of growth traits in Nellore sheep. Genetic estimation of growth traits in Nellore sheep revealed a heritability of 0.27 for body weight at 6 months of age in the model. Habtegiorgis K. et al. [16] Doyogena sheep early growth study yielded a heritability of 0.13 for body weight at 6 months of age. These findings are in agreement with the results of the present paper, but were greater than those in the genetic parameter estimation of early growth traits in Merino sheep by Gowane G. R. et al. [17]. The heritability of weekly weight at the age of 0.09 was reported in the estimation of genetic parameters for early growth traits of Merino sheep by Gowane G. R. et al. [17]. The slight difference in the results of the above studies is due to the difference in breeds and rearing conditions and other factors, and it also indicates that the heritability of the weight trait in Pishan Red sheep tends to be stabilized in the later stages of growth.

There was no estimate of maternal heritability at 12 months of age in Pishan Red sheep because at 12 months of age lambs are completely independent of the nursing influence of the ewe, nutritional intake, growth and development are independent and maternal effects (e.g., lactation behavior, etc.) on their body weights have basically disappeared; therefore, a model incorporating maternal heritability was not constructed in the present study, and maternal heritability was not estimated at this stage. The heritability of body weight of Pishan Red sheep at 12 months of age was 0.22 (medium heritability category), which was basically consistent with the results of Matebesi P. A. et al. [18] on Tygerhoek Merino flock sheep (0.33 at weekly age), and Naidoo P. et al. [19] on Merino sheep (0.31 at weekly age), suggesting that genetic transmission of body weight traits in Pishan Red sheep This value was lower than that of the genetic transmission of body weight at 2 months of age. This value was lower than the heritability of body weight at 2 months of age (0.69, high heritability) and 6 months of age (0.26, medium heritability), which further confirmed the pattern of increasing and then decreasing heritability of body weight in Pishan Red sheep, and also reflected that the influence of factors on body weight gradually increased with the growth of the lambs, and the dominant role of the genetic factors was weakened.

Heritability is a core indicator of the ability to transmit traits genetically and serves as an important basis for determining breeding methods and selection intensity. It is generally considered that heritability < 0.2 indicates low heritability, 0.2–0.4 medium heritability, and >0.4 high heritability. In the present study, the heritability of body weight at 0 months of age and the maternal heritability in Pishan Red sheep were 0.18 and 0.21, respectively, both falling into the medium heritability range. These results are consistent with those of Ren et al. [7] for Luzhong meat sheep (0.18 for direct heritability at birth, 0.20 for maternal heritability), El Fadili et al. [8] for Timahdit sheep (0.18 at birth), and Mandal et al. [9] for Muzaffarnagari sheep (0.21 for direct, 0.18 for maternal). However, they are lower than those reported by Habtegiorgis et al. [10] for Doyogena sheep (0.33 at birth) and Li et al. [11] for Alpine Merino sheep (0.30 at birth). These differences may be related to the genetic background of the breed, rearing conditions, and sample size.

For body weight at 2 months of age, the direct heritability and maternal heritability of Pishan Red sheep were 0.69 and 0.19, respectively, with direct heritability being a high-heritability trait and maternal heritability being medium to low. This result is significantly higher than those reported by Haile et al. [12] for Sabi sheep (weaning weight 0.13), Rashidi et al. [13] for Kermani sheep (weaning weight 0.27), and Hanford et al. [14] for Australian Merino sheep (weaning weight 0.10). The substantial difference between our result and the weaning weight heritability of most sheep breeds (typically 0.13–0.27) mainly stems from the breed characteristics of Pishan Red sheep, namely excellent year-round estrus and high ewe performance, which result in lamb growth during lactation being genetically dominated and maternal interference diminished. This reflects the breed’s characteristic of rapid early growth.

The heritability of body weight at 6 months of age in Pishan Red sheep was 0.26, which falls within the medium heritability range (h^2^ = 0.2–0.4). Maternal heritability was not calculated for this stage because, although lambs are significantly affected by maternal effects (e.g., milk production and lactation behavior) during the lactation period (0–2 months of age), by 6 months of age, lambs are fully weaned and have independent nutritional sources. Consequently, the influence of maternal effects on growth traits is substantially reduced. Therefore, the present study did not include a maternal heritability effect model for the 6-month stage, and maternal heritability was not estimated. Sila et al. [15] conducted a genetic estimation of growth traits in Nellore sheep and reported a heritability of 0.27 for body weight at 6 months of age. Habtegiorgis et al. [16], in their study on early growth of Doyogena sheep, found a heritability of 0.13 for body weight at 6 months. These findings are consistent with our results but are higher than those reported by Gowane et al. [17] in their genetic parameter estimation for early growth traits in Merino sheep, where the heritability of weekly weight at 6 months of age was 0.09. The slight differences among these studies are attributable to variations in breed, rearing conditions, and other factors, and also indicate that the heritability of body weight in Pishan Red sheep tends to stabilize during later growth stages.

Maternal heritability was not estimated at 12 months of age in Pishan Red sheep because, by this age, lambs are completely independent of the ewe’s nursing influence. Nutritional intake, growth, and development are fully autonomous, and maternal effects (e.g., lactation behavior) on body weight have essentially disappeared. Therefore, a model incorporating maternal heritability was not constructed for this stage. The heritability of body weight at 12 months of age was 0.22 (medium heritability category), which is broadly consistent with the results of Matebesi et al. [18] on Tygerhoek Merino flock sheep (0.33 at 12 months) and Naidoo et al. [19] on Merino sheep (0.31 at 12 months). This value is lower than the heritability of body weight at 2 months (0.69, high heritability) and 6 months (0.26, medium heritability), further confirming the pattern of an initial increase followed by a decrease in body weight heritability in Pishan Red sheep. It also reflects that, as lambs grow, the influence of environmental and other factors on body weight gradually increases, while the dominant role of genetic factors weakens.

#### 4.1.2. Analysis of Heritability of Body Measurements

In this study, among the body measurement traits of Pishan Red sheep at 0 months of age, the heritabilities of body height, body length, heart girth, chest width, chest depth, cannon circumference, and waist angle width were 0.09, 0.19, 0.30, 0.17, 0.15, 0.60, and 0.11, respectively. Among these, body height was a low-heritability trait, cannon circumference was a high-heritability trait, and the remaining traits fell into the medium-heritability category. The high heritability (0.35–0.71) of cannon circumference—a trait reflecting limb strength and development in Pishan Red sheep across all stages—is highly consistent with the dual-purpose (meat and fat) characteristics and high meat yield of this breed. This may be related to the strong stress resistance of Pishan Red sheep, the early maturity of traits (as indicated by the heritability of cannon circumference at 6 months of age), and the amount of exercise the sheep receive. Consequently, cannon circumference can be indirectly used as an auxiliary breeding index for meat production performance. Mandal et al. [9] reported that the direct heritabilities of body length, body height, and heart girth at the newborn stage were 0.14, 0.14, and 0.07, respectively. These values are similar to the heritability of body length at the newborn stage observed in the present study for Pishan Red sheep, whereas some differences were noted for body height and heart girth.

At 2 months of age, the heritabilities of body size traits were as follows: body weight 0.17, body height 0.37, body length 0.04, heart girth 0.22, chest depth 0.04, chest width 0.45, cannon circumference 0.26, and waist angle width 0.69. Among these, body weight, body length, and cannon circumference exhibited high heritability; body height and waist angle width showed medium heritability; and heart girth and chest depth had low heritability. In contrast, Mandal et al. [9] reported that the heritabilities of body length, body height, and heart girth in Muzaffarnagari sheep at the weaning stage were 0.12, 0.16, and 0.15, respectively, which differ to varying degrees from the corresponding trait heritabilities in Pishan Red sheep observed in this study. The primary reason for these differences in heritability estimates for body size traits at the same growth stage among different breeds is that the development of early growth traits in sheep is highly dependent on maternal effects. Factors such as ewe performance, body condition, and feeding management can significantly influence the expression of genetic effects for lamb lactation traits. Furthermore, there are fundamental differences in genetic background and breeding direction (meat and fat vs. local wool and meat) between Pishan Red sheep and Muzaffarnagari sheep, which also contribute to the divergence in heritability estimates. Notably, the heritability of 2-month body weight in Pishan Red sheep (0.69) was significantly higher than that reported for other sheep breeds (0.13–0.27). This uniqueness stems from the breed’s advantage of year-round estrus and excellent ewe performance, which results in lamb growth during lactation being dominated by genetic factors, thereby facilitating early and rapid genetic progress.

At 6 months of age, the heritabilities of body size traits in Pishan Red sheep were as follows: body height, 0.05; body length, 0.05; heart girth, 0.04; chest width, 0.48; chest depth, 0.16; cannon circumference, 0.71; and waist angle width, 0.08. Among these, chest width and cannon circumference were high-heritability traits; chest depth was a medium-heritability trait; and body height, body length, heart girth, and waist angle width were all low-heritability traits. A study on the genetic parameters of post-weaning Muzaffarnagari sheep by Mandal et al. [9] showed that the heritabilities of body height, body length, and heart girth at 6 months of age were 0.11, 0.14, and 0.14, respectively, which are higher than the corresponding estimates for Pishan Red sheep in the present study.

At 12 months of age (one year), the heritabilities of body size traits in Pishan Red sheep were as follows: body height, 0.48; body length, 0.46; heart girth, 0.28; chest width, 0.07; chest depth, 0.28; cannon circumference, 0.35; and waist angle width, 0.35. At this stage, body height and body length were high-heritability traits; chest width was a low-heritability trait; and heart girth, chest depth, cannon circumference, and waist angle width all fell into the medium-heritability category. Qin et al. [20] found that the heritabilities of body length, body height, chest depth, and heart girth were 0.05, 0.03, 0.11, and 0.53, respectively. Except for heart girth, these values were lower than those obtained in the present study. Mandal et al. [9] reported heritabilities of 0.14, 0.19, and 0.24 for body length, body height, and heart girth in Muzaffarnagari sheep, while Abbasi et al. [21] found heritabilities of 0.11, 0.21, and 0.17 for body length, heart girth, and body height in Makooei sheep. The heritabilities of heart girth in the two aforementioned studies are generally consistent with the results of the present study. The conclusion proposed by Ghafouri-Kesbi et al. [22]—that the heritability of body weight in sheep gradually increases with age—is highly consistent with the developmental trend of body weight heritability observed in Pishan Red sheep in this study. Minor differences between this study and previous studies are mainly attributable to variations in the selection and setting of fixed effects during the experimental process, inherent differences in genetic background and breeding direction among distinct sheep breeds, as well as differences in feeding conditions, management practices, and sample sizes, all of which can influence the estimation of genetic parameters. As a characteristic index of limb development and meat-fat production, the high heritability of cannon circumference in Pishan Red sheep provides an important basis for the synergistic breeding of meat quality and stress resistance in this breed.

### 4.2. Genetic Correlation Analysis of Growth Traits in Pishan Red Sheep

Genetic correlation is a core index for measuring the degree of genetic association among different traits at the gene level and can directly provide an important genetic basis for multi-trait collaborative breeding in meat sheep. The results showed that the ranges of genetic correlation between body weight and body size traits at 0, 2, 6, and 12 months of age were −0.15 to 0.84, −0.78 to 0.96, −0.32 to 0.70, and −0.04 to 0.87, respectively. The overall genetic correlations were predominantly positive, with only a small number of negative genetic correlations. As age increased, the fluctuation range of genetic correlations gradually narrowed, and the negative correlation effects progressively weakened. Negative correlations between certain traits may result from antagonistic interactions at different developmental stages and differences in energy allocation. Previous studies have shown similar genetic patterns to those observed in this study. Abbasi et al. [22] found that the genetic correlations between body size traits such as body length, body height, and heart girth and body weight ranged from 0.15 to 0.99. Dige et al. [23] pointed out, in a study on weight prediction and genetic parameters in goats, that genetic correlations between body size traits before and after weaning ranged from 0.09 to 0.99 and showed a trend of gradually increasing with age. Ghavi Hossein-Zadeh [24] found that direct genetic correlations among morphological traits ranged from −0.21 to 0.67, and that body weight exhibited positive genetic correlations with all body size traits. There are slight differences between the present study and the aforementioned results. These differences are mainly related to the genetic background and breeding direction of the research subjects. At the same time, variations in sample size, feeding intensity, accuracy, and standardization of data collection, as well as differences in the prioritization of nutrient allocation among organs and traits at different growth stages of sheep, can all influence the estimation of genetic correlations. The positive genetic correlation characteristics between body weight and body size traits at each month of age in Pishan Red sheep provide a solid genetic basis for indirect selection and multi-trait synergistic selection in the breeding practice of this breed. Traits that show significant positive genetic correlations at each stage can be included as auxiliary selection indicators in the breeding and selection practices for Pishan Red sheep to improve breeding and selection efficiency.

### 4.3. Phenotypic Correlation Analysis of Growth Traits of Pishan Red Sheep

The results of this study showed that the phenotypic correlations between body weight and body size traits of Pishan Red sheep at different months of age exhibited distinct stage characteristics. The ranges of phenotypic correlation coefficients at 0, 2, 6, and 12 months of age were 8.2 × 10^−3^–0.32, 0.036–0.44, −0.13–0.54, and 0.28–0.72, respectively. The overall trend showed a gradual increase with age, with phenotypic correlations becoming significantly stronger during the later growth stage and reaching a peak at 12 months of age. At the same time, a small number of negative phenotypic correlations appeared at 6 months of age, while the remaining age stages were dominated by positive phenotypic correlations. Previous studies on other sheep breeds have also reported similar phenotypic correlation patterns. Gad [25] found that the phenotypic correlation coefficients between body weight and body size traits such as body length, body height, and heart girth in BARKI sheep ranged from 0.43 to 0.56. The study by Jafari and Hashemi [26] in Makuie sheep showed that the phenotypic correlation coefficients between body weight and body size traits ranged from 0.32 to 0.90, which are slightly higher than the corresponding estimates for Pishan Red sheep in the present study. The differences between this study and the aforementioned results are mainly attributable to inherent differences in the genetic background and growth characteristics of different sheep breeds. Additionally, variations in the geographical conditions of the breeding areas, differences in feeding and management practices, as well as differences in sample size and population structure, all have a certain impact on the estimation of phenotypic correlations. Nevertheless, the overall differences in phenotypic correlation coefficients are small, which further confirms that a positive phenotypic correlation between body weight and body size traits is a common growth pattern in sheep.

The experimental sample for this study was sourced exclusively from a single farm in the Hotan Prefecture of Xinjiang. The estimates of genetic parameters did not account for the effects of different rearing environments or breeds, and their representativeness requires further validation. In future studies, the scope of sample collection could be expanded to include Pishan Red sheep populations from different farming systems and regions across the Hotan Prefecture, enabling the estimation of genetic parameters under multiple environmental conditions to further validate the stability and applicability of the genetic parameters obtained in this study.

## 5. Conclusions

For the first time, this study systematically estimated the genetic parameters of body weight and body size of Pishan Red sheep at the age of 0–12 months, and clarified that body weight (high heritability 0.69 at the age of 2 months) and pipe circumference (high heritability 0.35–0.71 at the whole stage) are the early selection criteria of Pishan Red sheep. Core indicators, it is suggested that in breeding practice, 2 months of age is the key window period for weight selection, and giving priority to individuals with larger body weight and pipe circumference at the age of 2 months can significantly improve the selection response and provide technical support for improving the quality and efficiency of the meat sheep industry in southern Xinjiang, the research results provide the first local data support for the genetic improvement of this breed.

## Figures and Tables

**Table 1:** Division of fixed effects.

Level	Factors		
	Year of Birth	Gender	Birth Type
1	2021	Rams	Single lamb
2	2022	Ewes	Double lamb
3	2023		Three lambs
4	2024		Four lambs

**Table 2:** Modeling of genetic parameters using DMUv6-R5-2-EM software.

Prepare Documents	Pedigree File	Data Record File (Phenotypic Data)	DIR File
Fixed effect	Individual number, father number, mother number, date of birth	Year of birth, type of birth, gender	Individual number, father number, mother number, date of birth, year of birth, type of birth, gender
Traits		Body weight, body height, body length, chest circumference, chest width, chest depth, tube circumference, and waist angle width of 0~12 months old were measured	
Animal model	y = Xb + Z1α + Z2m + e; y = Xb + Z1α + e		
Formula for calculating genetic parameters	Direct heritability h^2^_a_ = σ_a_^2^/σ_p_^2^ Maternal heritability h^2^_m_ = σ_m_^2^/σ_p_^2^		

Note: In the formula, y = a trait value vector of the target; b = fixed effect vector; α = individual genetic vector effect; m = maternal additive genetic effect vector; e = random residual vector; X, Z1, and Z2 correspond to the design matrix of fixed effect vector, additive genetic effect vector, and maternal additive genetic effect vector.

**Table 3:** Variance (ANOVA) for body weight and size indexes of Pishan Red sheep.

Traits	Age (Months)	Gender (F Value)	Birth Type (F Value)	Year of Birth (F Value)
W	0	46.12 **	8501.77 **	362.85 **
	2	66.47 **	2109.64 **	254.10 **
	6	1260.04 **	795.32 **	686.17 **
	12	8793.20 **	455.38 **	310.90 **
BH	0	28.11 **	72.92 **	22.83 **
	2	445.62 **	25.21 **	37.02 **
	6	412.71 **	103.10 **	177.02 **
	12	2176.10 **	42.24 **	197.35 **
BL	0	35.33 **	135.79 **	55.92 **
	2	21.88 **	5.14 **	89.17 **
	6	102.36 **	61.21 **	100.96 **
	12	1387.80 **	29.47 **	105.57 **
CG	0	142.64 **	157.66 **	38.38 **
	2	52.61 **	13.36 **	66.25 **
	6	904.02 **	135.07 **	323.82 **
	12	421.73 **	151.44 **	105.50 **
CW	0	7.28 **	13.63 **	52.08 **
	2	8.36 *	3.00 *	144.92 **
	6	211.71 **	87.44 **	153.52 **
	12	705.58 **	75.11 **	98.14 **
CD	0	48.39 **	11.12 **	11.93 **
	2	42.96 **	4.99 *	109.36 **
	6	634.85 **	70.52 **	953.56 **
	12	552.58 **	130.44 **	302.77 **
PG	0	1.72	121.84 **	608.85 **
	2	17.46 **	5.74 **	65.30 **
	6	92.31 **	5.97 *	128.91 **
	12	42.71 **	20.99 **	59.82 **
HW	0	65.77 **	33.10 **	15.53 **
	2	7.98 *	4.51 *	34.82 **
	6	384.64 **	25.94 **	472.67 **
	12	1649.57 **	4.18 *	80.05 **

Note: * denotes *p* < 0.05, ** denotes *p* < 0.01, unified F-value labeling; W stands for body weight, BH for body height, BL for body length, CG for chest circumference, CW for chest width, CD for chest depth, PG for cannon bone circumference, and HW for loin width. The same applies to the table below.

**Table 4:** Estimates of variance components and heritability of growth traits in Pishan Red sheep.

Age (Months)	Traits	σ_a_^2^	Cov	σ_m_^2^	σ_e_^2^	σ_p_^2^	h^2^	h^2^_m_
0 months	W	0.31	0.71	0.33	0.14	1.6	0.18	0.21
	BH	0.88	−1.51	3.09	7.68	10.13	0.09	0.3
	BL	1.85	−1.57	2.19	7.15	9.62	0.19	0.23
	CG	3.41	−2.83	4.86	5.81	11.24	0.3	0.43
	CW	0.35	−0.18	0.96	0.86	1.99	0.17	0.48
	CD	0.44	−0.42	0.63	2.17	2.82	0.15	0.22
	PG	0.43	−0.35	0.46	0.18	0.72	0.6	0.64
	HW	0.1	0.54	0.09	0.12	0.92	0.11	0.09
2 months	W	0.7	−0.23	0.19	0.34	1	0.69	0.19
	BH	1.62	−0.79	1.16	7.53	9.52	0.17	0.12
	BL	2.36	−0.7	0.5	4.13	6.3	0.37	0.07
	CG	1	−0.59	3.45	24.19	28.05	0.04	0.13
	CW	0.59	−0.97	0.16	2.87	2.65	0.22	0.06
	CD	0.23	−0.22	0.52	4.88	5.41	0.04	0.09
	PG	0.18	−0.53	0.2	0.55	0.39	0.45	0.51
	HW	0.72	−0.68	0.24	2.47	2.75	0.26	0.09
6 months	W	0.39	−0.51		1.63	1.5	0.26	
	BH	0.48	−0.82		9.25	8.9	0.05	
	BL	0.37	−0.23		6.96	7.1	0.05	
	CG	0.24	−0.74		6.5	6	0.04	
	CW	0.77	−0.28		1.12	1.61	0.48	
	CD	0.32	−0.73		2.43	2.02	0.16	
	PG	0.77	0.15		0.17	1.1	0.71	
	HW	0.14	0.15		1.41	1.7	0.08	
12 months	W	1.11	−1.31		5.32	5.12	0.22	
	BH	4.17	−3.62		8.21	8.76	0.48	
	BL	5.39	−4.88		11.09	11.6	0.46	
	CG	11.09	−3.25		31.56	39.4	0.28	
	CW	0.46	−0.87		6.49	6.08	0.07	
	CD	1.65	−1.2		5.35	5.8	0.28	
	PG	0.27	0.18		0.32	0.77	0.35	
	HW	0.27	0.18		0.32	0.77	0.35	

Note: σ_a_^2^ = additive genetic variance; Cov = covariance between direct additive genetic effect and maternal additive genetic effect; σ_m_^2^ = maternal genetic variance; σ_e_^2^ = residual variance; σ_p_^2^ = phenotypic variance; h^2^ = heritability; h^2^_m_ = maternal inheritance.

**Table 5:** Genetic correlation of growth traits in Pishan Red sheep.

Age (Months)	Traits	BH	BL	CG	CW	CD	PG	HW
0 months	W	0.19 ± 0.01	0.11 ± 0.04	0.07 ± 0.03	0.21 ± 0.08	0.09 ± 0.05	0.06 ± 0.03	−0.02 ± 0.1
	BH		0.20 ± 0.13	0.29 ± 0.11	0.43 ± 0.09	0.31 ± 0.18	0.12 ± 0.03	−0.02 ± 0.01
	BL			0.52 ± 0.08	0.21 ± 0.05	0.07 ± 0.02	0.11 ± 0.02	−0.08 ± 0.05
	CG				0.72 ± 0.25	0.29 ± 0.15	0.84 ± 0.15	0.34 ± 0.04
	CW					0.34 ± 0.14	0.75 ± 0.25	−0.02 ± 0.01
	CD						−0.15 ± 0.16	−0.01 ± 0.11
	PG							−0.03 ± 0.06
	HW							
2 months	W	0.15 ± 0.06	−0.07 ± 0.12	0.86 ± 0.18	0.35 ± 0.07	0.09 ± 0.04	0.34 ± 0.20	0.28 ± 0.07
	BH		0.44 ± 0.13	−0.05 ± 0.08	0.38 ± 0.04	0.73 ± 0.20	0.38 ± 0.17	0.15 ± 0.01
	BL			0.55 ± 0.07	0.64 ± 0.09	0.22 ± 0.08	0.58 ± 0.09	0.94 ± 0.04
	CG				0.68 ± 0.07	0.50 ± 0.09	0.47 ± 0.15	0.96 ± 0.04
	CW					−0.22 ± 0.06	0.75 ± 0.08	−0.48 ± 0.11
	CD						−0.39 ± 0.02	−0.39 ± 0.13
	PG							−0.78 ± 0.11
	HW							
6 months	W	0.16 ± 0.06	0.18 ± 0.05	0.18 ± 0.05	0.40 ± 0.05	0.18 ± 0.06	0.39 ± 0.06	0.17 ± 0.06
	BH		0.61 ± 0.08	0.70 ± 0.07	0.52 ± 0.09	0.49 ± 0.09	0.12 ± 0.01	0.35 ± 0.10
	BL			0.54 ± 0.08	0.51 ± 0.08	0.25 ± 0.09	0.45 ± 0.10	0.14 ± 0.10
	CG				0.45 ± 0.08	0.49 ± 0.08	0.10 ± 0.08	0.17 ± 0.05
	CW					0.55 ± 0.08	0.17 ± 0.10	0.39 ± 0.09
	CD						−0.26 ± 0.10	0.42 ± 0.09
	PG							−0.32 ± 0.10
	HW							
12 months	W	0.28 ± 0.08	0.26 ± 0.07	0.30 ± 0.06	0.09 ± 0.06	0.34 ± 0.08	0.25 ± 0.07	0.13 ± 0.09
	BH		0.87 ± 0.07	0.59 ± 0.09	0.40 ± 0.11	0.72 ± 0.09	0.58 ± 0.11	0.22 ± 0.15
	BL			0.78 ± 0.07	0.59 ± 0.09	0.74 ± 0.10	0.70 ± 0.10	0.20 ± 0.15
	CG				0.57 ± 0.08	0.67 ± 0.08	0.61 ± 0.09	0.27 ± 0.13
	CW					0.43 ± 0.10	0.61 ± 0.10	−0.04 ± 0.15
	CD						0.57 ± 0.11	0.16 ± 0.14
	PG							0.18 ± 0.15
	HW							

## Data Availability

The original contributions presented in this study are included in the article/Supplementary Material. Further inquiries can be directed to the corresponding author.

## Supplementary Materials

The following supporting information can be downloaded at: https://www.mdpi.com/article/10.3390/ani16101569/s1, Table S1: Descriptive Statistics on Body Measurements and Body Weight of Pishan Red Sheep at 0 Months of Age; Table S2: Descriptive Statistics on Body Measurements and Body Weight of 2-Month-Old Pishan Red Sheep; Table S3: Descriptive Statistics of Body Measurements and Body Weight of 6-Month-Old Pishan Red Sheep; Table S4: Descriptive Statistics on Body Measurements and Body Weight of 12-Month-Old Pishan Red Sheep.
