# Supplementary material for: Estimation of Genetic Parameters of Body Weight and Body Size in Different Stages of Pishan Red Sheep
Source: Animals (Basel). 2026 May 21;16(10):1569. doi: 10.3390/ani16101569 (PMC13203375; doi:10.3390/ani16101569)
Supplement: Supplementary file 1 [file animals-16-01569-s001.zip › animals-4277222-supplementary.pdf]

Supplementary Tables S1 – S4

Descriptive Statistics on Body Weight and Body Measurements of Pishan Red Sheep at Different Stages

As shown in Tables S1 – S4, the coefficients of variation for body weight, height, body length, chest circumference, chest width, chest depth, cannon bone circumference, and loin width in Pishan Red sheep were highest at 0 months of age, followed by those at 2 months of age. Throughout this period, the coefficients of variation were consistently higher in rams than in ewes. These findings indicate that there is substantial selection potential for body weight and body measurements in Pishan Red sheep lambs at 0 and 2 months of age. During the fetal stage, providing ewes with a high-nutrient feed can help achieve the ideal birth weight and body measurements in lambs. At weaning, in addition to improving the feed provided to ewes, it is also recommended to offer starter feed suitable for lamb growth and development from birth until approximately two weeks of age. This strategy can further enhance lamb weight and body measurements at 2 months of age.

Table S1 Descriptive Statistics on Body Measurements and Body Weight of Pishan Red Sheep at 0 Months of Age.

Gender	Traits	Quantity	minimum	maximum	average	SD	variance	CV.%
Ram	W,kg	2767	1.90	5.70	3.72	0.63	0.39	16.87
Ewe		3069	1.85	5.75	3.67	0.91	0.83	24.77
Ram	BH,cm	2767	28.89	41.09	34.39	1.96	3.84	5.7
Ewe		3069	21.6	43.6	33.28	3.51	12.29	10.53
Ram	BL,cm	2767	20.3	36.7	31.06	2.45	6.01	7.89
Ewe		3069	20.1	42.2	31.29	3.99	15.89	12.74
Ram	CG,cm	2767	28.7	41.6	35.17	2.1	4.43	5.98
Ewe		3069	21.1	70.7	34.32	3.86	14.91	11.25
Ram	CW,cm	2767	10.14	7.97	10.14	0.93	0.87	11.67
Ewe		3069	10	7.9	10	0.88	0.78	11.18
Ram	CD,cm	2767	9.94	18.34	13.95	1.6	2.56	11.48
Ewe		3069	9.6	18	13.79	1.56	2.43	11.32
Ram	PG,cm	2767	5.26	7.89	5.54	0.41	0.17	7.46
Ewe		3069	5.11	7.5	5.81	0.49	0.24	8.43
Ram	HW,cm	2767	5.08	9.48	7.38	0.96	0.92	13.01
Ewe		3069	5.04	9.34	7.16	0.93	0.87	13.03

Table S2 Descriptive Statistics on Body Measurements and Body Weight of 2-Month-Old Pishan Red Sheep.

Gender	Traits	Quantity	minimum	maximum	average	SD	variance	CV.%
Ram	W,kg	2102	10.35	20.95	17.31	2.31	5.33	13.34
Ewe		2683	10.40	19.30	16.7	2.09	4.39	12.54
Ram	BH,cm	2102	48.71	60.05	53.05	2.91	8.45	5.48
Ewe		2683	46.49	56.60	51.34	2.75	7.55	5.35
Ram	BL,cm	2102	40.54	50.03	43.65	2.15	4.40	4.80
Ewe		2683	46.27	43.52	1.83	3.35	4.20	5.91
Ram	CG,cm	2102	51.49	72.20	60.37	3.29	10.85	5.46
Ewe		2683	51.15	67.95	58.5	4.26	18.13	7.28
Ram	CW,cm	2102	16.67	13.19	16.24	1.64	2.68	12.42
Ewe		2683	16.41	12.68	16.41	1.73	2.99	13.64
Ram	CD,cm	2102	16.52	29.50	23.49	2.39	5.71	10.17

Ewe		2683	17.75	27.75	22.78	2.06	4.25	9.05
Ram	PG,cm	2102	6.31	8.80	7.48	0.59	0.34	7.85
Ewe		2683	6.12	8.50	7.21	0.59	0.35	8.25
Ram	HW,cm	2102	10.91	17.00	12.93	1.95	3.61	14.69
Ewe		2683	10.05	16.05	12.52	1.53	2.33	12.18

Table S3 Descriptive Statistics of Body Measurements and Body Weight of 6-Month-Old Pishan Red Sheep.

**Gender**	**Traits**	**Quantity**	**minimum**	**maximum**	**average**	**SD**	**variance**	**CV,%**
Ram	W,kg	1353	26.40	40.50	35.44	3.81	14.54	10.76
Ewe		2338	27.10	36.95	32.28	2.67	7.14	8.28
Ram	BH,cm	1353	55.50	70.50	65.79	3.93	15.46	5.98
Ewe		2338	53.90	68.90	63.88	3.26	10.61	5.10
Ram	BL,cm	1353	47.20	59.20	55.43	2.92	8.52	5.27
Ewe		2338	48.70	59.70	53.39	3.09	9.56	5.79
Ram	CG,cm	1353	69.00	83.00	76.54	4.31	18.60	5.63
Ewe		2338	66.00	79.50	73.15	3.95	15.60	5.40
Ram	CW,cm	1353	22.50	18.80	22.50	1.51	2.29	8.05
Ewe		2338	22.80	17.68	22.80	1.75	3.05	9.87
Ram	CD,cm	1353	25.00	30.00	28.77	1.05	1.10	3.65
Ewe		2338	24.10	32.90	27.57	2.04	4.18	7.42
Ram	PG,cm	1353	7.20	9.00	8.20	0.38	0.14	4.60
Ewe		2338	7.10	8.70	8.09	0.52	0.27	6.47
Ram	HW,cm	1353	16.50	22.50	18.28	0.82	0.67	4.48
Ewe		2338	15.00	22.00	17.29	1.14	1.30	6.59

Table S4 Descriptive Statistics on Body Measurements and Body Weight of 12-Month-Old Pishan Red Sheep

**Gender**	**Traits**	**Quantity**	**minimum**	**maximum**	**average**	**SD**	**variance**	**CV.%**
Ram	W,kg	565	49.5	76.80	68.72	5.92	35.1	8.62
Ewe		1931	39.00	58.00	49.48	5.55	30.81	11.22
Ram	BH,cm	565	67.52	84.00	79.59	2.93	8.57	3.68
Ewe		1931	65.5	81.50	69.66	3.55	12.61	5.10
Ram	BL,cm	565	66.00	75.00	69.50	2.79	7.76	4.01
Ewe		1931	57.00	72.00	59.91	2.87	8.22	4.78
Ram	CG,cm	565	76.00	114.00	90.16	8.58	73.54	9.51
Ewe		1931	70.00	111.00	82.52	6.83	46.68	8.28
Ram	CW,cm	565	28.50	24.10	28.50	1.44	2.06	5.96
Ewe		1931	32.00	21.18	32.00	2.72	7.38	12.83
Ram	CD,cm	565	33.50	42.50	36.42	2.28	5.19	6.25
Ewe		1931	27.00	42.00	32.30	2.85	8.13	8.83
Ram	PG,cm	565	7.40	9.90	8.61	0.82	0.67	9.49
Ewe		1931	7.70	9.90	8.42	0.63	0.40	7.51
Ram	HW,cm	565	20.00	29.50	22.68	1.98	3.91	8.71
Ewe		1931	16.50	25.50	18.51	1.40	1.97	7.58
